# Genetic inhibition of autophagy promotes p53 loss-of-heterozygosity and tumorigenesis

**DOI:** 10.18632/oncotarget.12084

**Published:** 2016-09-16

**Authors:** Eunmyong Lee, Yongjie Wei, Zhongju Zou, Kathryn Tucker, Dinesh Rakheja, Beth Levine, James F. Amatruda

**Affiliations:** ^1^ Department of Internal Medicine, University of Texas Southwestern Medical Center, Dallas, Texas, USA; ^2^ Center for Autophagy Research, University of Texas Southwestern Medical Center, Dallas, Texas, USA; ^3^ Department of Pediatrics, University of Texas Southwestern Medical Center, Dallas, Texas, USA; ^4^ Department of Pathology, University of Texas Southwestern Medical Center, Dallas, Texas, USA; ^5^ Department of Microbiology, University of Texas Southwestern Medical Center, Dallas, Texas, USA; ^6^ Howard Hughes Medical Institute, University of Texas Southwestern Medical Center, Dallas, Texas, USA; ^7^ Department of Molecular Biology, University of Texas Southwestern Medical Center, Dallas, Texas, USA

**Keywords:** Autophagy, p53, MPNST, loss-of-heterozygosity, zebrafish

## Abstract

Autophagy is an evolutionarily conserved lysosomal degradation pathway that plays an essential role in enabling eukaryotic organisms to adapt to nutrient deprivation and other forms of environmental stress. In metazoan organisms, autophagy is essential for differentiation and normal development; however, whether the autophagy pathway promotes or inhibits tumorigenesis is controversial, and the possible mechanisms linking defective autophagy to cancer remain unclear. To determine if autophagy is important for tumor suppression, we inhibited autophagy in transgenic zebrafish via stable, tissue-specific expression of a dominant-negative autophagy protein Atg5^K130R^. In heterozygous *tp53* mutants, expression of dominant-negative *atg5^K130R^* increased tumor incidence and decreased tumor latency compared to non-transgenic heterozygous *tp53* mutant controls. In a *tp53-*deficient background, Tg(*mitfa:atg5^K130R^*) mutantsdeveloped malignant peripheral nerve sheath tumors (MPNSTs), neuroendocrine tumors and small-cell tumors. Expression of a Sox10-dependent GFP transgene in the tumors demonstrated their origin from neural crest cells, lending support to a model in which *mitfa*-expressing cells can arise from *sox10*+ Schwann cell precursors. Tumors from the transgenic animals exhibited increased DNA damage and loss-of-heterozygosity of *tp53*. Taken together, our data indicate that genetic inhibition of autophagy promotes tumorigenesis in *tp53* mutant zebrafish, and suggest a possible role for autophagy in the regulation of genome stability during oncogenesis.

## INTRODUCTION

Autophagy is a highly conserved cellular degradation pathway that mediates the degradation of macromolecules and organelles by the lysosome [1]. Serving as an intracellular “recycling” pathway, autophagy ensures the continued availability of building blocks for macromolecular synthesis during periods of cellular stress and nutrient deprivation. Autophagy also exerts a quality control function by removing misfolded or unfolded proteins and damaged organelles. In light of these many important functions, it is not surprising that autophagy plays an essential role in normal physiology and protection against diseases, including neurodegenerative diseases, infection and aging [2, 3].

The role of autophagy in cancer remains controversial, with studies suggesting both pro- and anti-tumor effects of autophagy genes [4]. Supporting a tumor-suppressive effect of autophagy, several studies indicate that autophagy prevents tumor initiation. *BECLIN 1* (*BECN1*) is frequently monoallelically deleted in human breast and ovarian cancers [5, 6]; *Becn1* heterozygous-deficient mice have an increased frequency of spontaneous malignancies including lymphomas, liver, lung and breast tumors [7-9]; and decreased *BECN1* expression is associated with poor outcomes in human breast cancer [10]. Mice deficient in *Atg4C* have increased chemically-induced fibrosarcomas [11], and *Bif-1* deletion in mice results in an increased frequency of spontaneous lymphomas and solid tumors [12]. The anticancer effects of tumor-suppressor genes such as *PTEN, LKB1* and *TSC1/TSC2* derive, at least in part, from their regulation of PI3K-mTOR signaling, which normally opposes the autophagy pathway. Consistent with this idea, oncogenes such as AKT, EGFR and BCL2 may promote tumorigenesis by inhibiting autophagy, either through a positive effect on mTOR signaling (reviewed in [13]) or directly *via* effects on Beclin 1 [14-16].

On the other hand, several studies suggest that the autophagy pathway can promote the survival of cancer cells under conditions of environmental stress. Inhibition of autophagy by deletion *FIP200* (also known as *RB1CC1*) suppresses the development of mammary tumors in a polyoma Middle T-driven breast cancer model [17]. Autophagy promotes survival and increases tumorigenesis in mutant *Kras*-expressing cells [18-20], and *Atg7* deletion in mice suppresses progression of Kras and Braf^V600E^ induced lung cancers and Kras driven pancreatic carcinoma [21-23]. Beclin 1 appears to be required for maintenance of a cancer stem cell population in breast cancers [24]. Taken together, these studies suggest a model whereby autophagy plays different roles in different stages of tumorigenesis. Autophagy may oppose the initiation of tumors by removing damaged organelles and proteins from the cytoplasm, exerting quality control and avoiding the generation of reactive oxygen species (ROS) by damaged mitochondria. Once a tumor is established however, autophagy may promote cancer cell survival in the face of stresses caused by rapid growth and the metabolic effects of oncogenes. In addition to different roles in different stages of tumorigenesis, autophagy may have different functions in the context of different tumor mutational status; for example, although loss of autophagy delays tumorigenesis in Kras-driven pancreatic tumors, it accelerates tumorigenesis in Kras-driven pancreatic tumors with concurrent p53 mutation [22].

To further address the role of autophagy in tumor development in an *in vivo* model system and to probe the interrelationship of autophagy and p53 deficiency, we inhibited autophagy in a tissue-specific fashion in zebrafish by expressing a dominant-negative form of the autophagy gene *atg5* (*atg5^K130R^*) from the well-characterized microphthalmia-associated transcription factor a (*mitfa*) promoter. *mitfa* is expressed in the developing neural crest and in melanocytes [25], and a previous study showed that transgenic expression of mutant BRAF^V600E^ kinase under the control of the *mitfa* promoter leads to melanoma in p53-mutant fish [26]. Fish stably expressing the *mitfa:*a*tg5^K130R^* transgene in the p53 mutant background developed several types of tumors including small round cell tumors, neuroendocrine tumors and malignant peripheral nerve sheath tumors (MPNSTs). Compared to control heterozygous *tp53M^214K/+^* heterozygotes, Tg(*mitfa:*a*tg5^K130R^*); *tp53M^214K/+^* fish displayed decreased latency and increased incidence of MPNSTs. Coexpression of a *sox10*-*eGFP* transgene demonstrated the neural crest origin of the tumors. Moreover, expression of Atg5^K130R^ caused increased DNA damage and accelerated loss-of-heterozygosity (LOH) of p53 in the *p53^M214K/+^* background. Taken together, these results suggest that autophagy acts as a barrier to tumor formation, and that defective autophagy may contribute to genome instability and tumorigenesis.

## RESULTS

### Expression of dominant-negative autophagy gene, *atg5^K130R^* impairs autophagy in zebrafish

**Figure 1:**
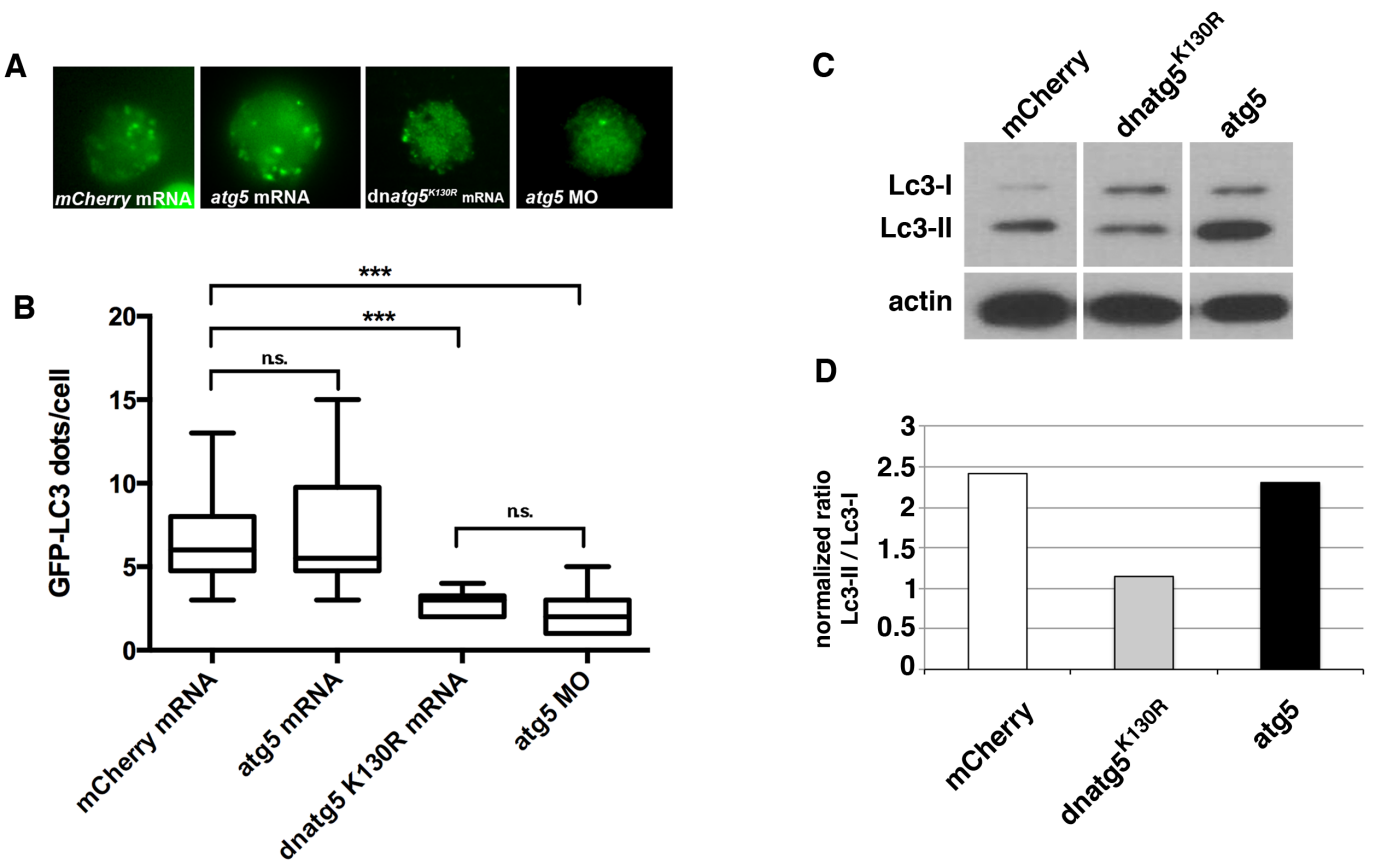
Autophagy is inhibited by expression of *atg5^K130R^* mRNA **A.**-**B.** Representative images **A.** and quantification **B.** of GFP-Lc3 puncta in primary cells prepared from blastula-stage Tg(*cmv:GFP-LC3*) embryos injected with mRNA encoding *mCherry* control, wildtype *atg5* or dominant-negative *atg5^K130R^*, or with a morpholino targeting *atg5*. At least 15 cells/group were scored. Bars represent mean ± SEM of 50 to 100 cells per group. Similar results were observed in three independent experiments. ***P < 0.001 for *atg5^K130R^* mRNA- or morpholino-injected embryos *versus mCherry* mRNA-injected embryos; two-tailed *t*-test. **C.** Immunoblot analysis of Lc3 in primary cell lysates from embryos injected at the one-cell stage with *in vitro* transcribed *mCherry*, *atg5^K130R^* or *atg5* mRNA. Actin is shown as a loading control. **D.** Quantification of immunoblot.

Previous studies have established that expression of Atg5 in which lysine 130 is mutated to arginine inhibits autophagy *via* a dominant-negative mechanism [27-30]. To confirm that the expression of Atg5^K130R^ inhibits autophagy in zebrafish *in vivo*,** we took advantage of a Tg(*cmv:GFP-lc3*)** autophagy reporter line [31]. During autophagy induction, Lc3-I (diffusely distributed in the cytoplasm) is conjugated with phosphatidylethanolamine to form Lc3-II, which stably associates with the autophagosomal membrane. GFP-tagged Lc3-II accumulates in punctate structures (autophagosomes), serving as a useful *in vivo* marker of autophagy [32]. We injected mRNA encoding mCherry, zebrafish *atg5* or dominant-negative *atg5^K130R^* mRNA, or a morpholino directed against *atg5* that we previously validated [33] into one-cell stage *Tg(CMV:GFP-Lc3)* embryos. We prepared primary gastrula-stage cultures from the injected embryos and used fluorescence microscopy to quantify the number of GFP-Lc3 puncta. Compared to *mCherry* or *atg5*, *atg5^K130R^* mRNA significantly reduced the number of GFP-LC3 puncta, similar to the effect of the *atg5* morpholino, indicating inhibition of autophagosome formation by expression of Atg5^K130R^ *in vivo* (Figure 1A, 1B).

We further confirmed that the expression of Atg5^K130R^ inhibits autophagy by immunoblot analysis of Lc3 in lysates prepared from embryos injected with the mRNAs. *atg5^K130R^* mRNA- injected embryo lysates showed reduced levels of Lc3-II compared to control *atg5* or *mcherry* mRNA-injected embryos, suggesting that autophagy is inhibited by the expression of Atg5^K130R^ (Figure 1C, 1D). In summary, the overexpression of *atg5^K130R^* RNA efficiently inhibited autophagy in zebrafish *in vivo*.

### Stable expression of *mitfa*: *atg5^K130R^* results in tumors in a *tp53^M214K/+^* background

Having shown that expression of Atg5^K130R^ inhibits autophagy in zebrafish embryos, we used this approach to study the role of autophagy in tumor suppression. To investigate the role of autophagy in tumor suppression, we expressed *atg5^K130R^* from the *mitfa* promoter. During zebrafish development, Mitf is expressed in neural crest and melanocytes and has been implicated as a key regulator in pigment cell development [25]. A previous report showed that transgenic zebrafish expressing mutant *BRAF^V600E^* driven by the *mitfa* promoter formed fish nevi and developed melanomas in a p53-mutant background [26].

**Figure 2:**
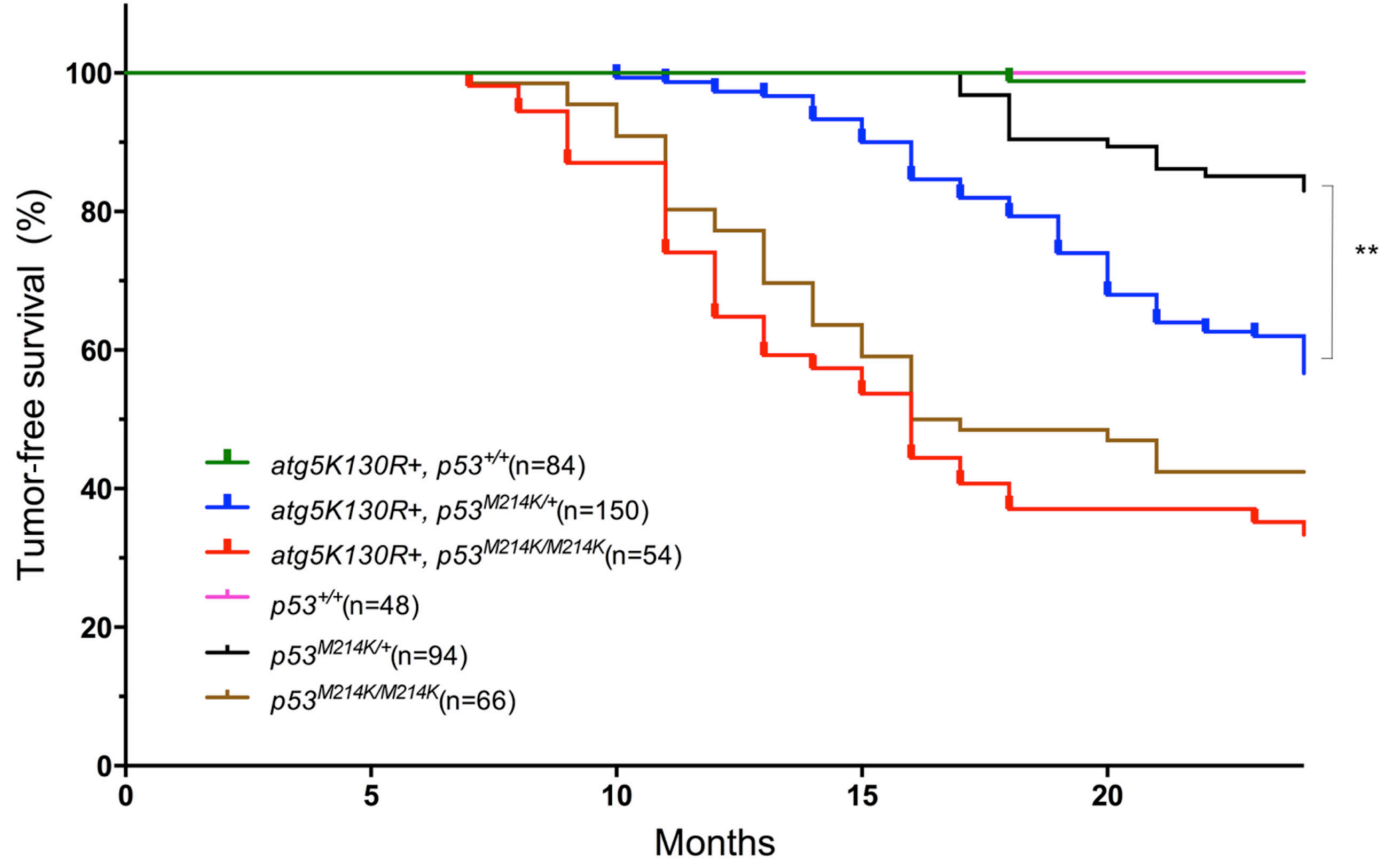
Atg5^K130R^ accelerates tumor development in the p53-mutant background Tumor-free survival for each indicated fish genotype is shown. Numbers in parentheses indicate total number of animals observed per genotype. **p < 0.001 for Tg(*mitfa:atg5^K130R^*)*; p53^M214K/+^ vs* non-transgenic *tp53^M214K/+^* (Log-rank (Mantel-Cox) test).

We used the *Tol2* transposon system [34] to generate genomic insertions of the *mitfa:atg5^K130R^* transgene, and identified founders by PCR analysis of F1 progeny (Supplementary Figure 1). Stable transgenics were raised to adulthood and monitored for tumor onset. Previous reports showed that chimeric knockout of the autophagy gene *Atg5* resulted in hepatomas in mice [35]. However, transgenic fish expressing Atg5^K130R^ did not develop tumors. We suspected that inhibition of autophagy alone might be insufficient to cause tumors, and therefore tested the effect of the transgene in the sensitized *tp53*-mutant background. Homozygous mutant *tp53^M214K^* fish were previously shown to have defects in the apoptotic response to γ-irradiation, as well as increased tumor susceptibility. The most common tumors in these fish are MPNSTs [36]. In our cohort, 55% of *tp53^M214K/M214K^* homozygotes developed MPNSTs beginning at 7 months of age (median age of onset 16.5 months); *tp53^M214K/+^* heterozygotes exhibited lower incidence (17%) and longer latency (median age of onset 20 months) (Figure 2), consistent with previous reports [36].

Transgenic expression of the dominant-negative autophagy construct increased tumor incidence and decreased latency in p53-deficient fish (Figure 2). In the p53-heterozygous background; nearly 40% of Tg(*mitfa:atg5^K130R^*)*;tp53^M214K/+^* heterozygotes developed tumors, with a median age of onset of 17 months. (*p* < 0.0001 for comparison to non-transgenic *tp53^M214K/+^* fish by log-rank test). Expression of Atg5^K130R^ in a *tp53^M214K/M214K^* background caused slightly earlier and higher tumor incidence compared to *tp53^M214K/M214K^* homozygotes (60% incidence: median age of onset 16 months), but the difference did not reach statistical significance (Figure 2). To verify that the tumors expressed the *atg5^K130R^* transgene, we prepared short-term primary cell cultures of the tumors (to enrich for tumor cells and allow for depletion of nonproliferating normal cells), purified RNA from the cells and performed RT-PCR with primers specific for *atg5*. This assay validated expression of the *atg5^K130R^* transgene in the tumors (Supplementary Figure 1).

For consistency, in these experiments all Tg(*mitfa:atg5^K130R^*) animals were derived from a single founder. To confirm these results, and to ensure that the effect of autophagy inhibition on tumor incidence was not due to unintended consequences of transgene genomic insertion, we generated an independent transgenic line in which the *mitfa* promoter drives expression of a dominant-negative form of the autophagy protein Atg4b [*Tg(mitfa:atg4b^C74A^)*]. Mutation of this conserved cysteine residue to alanine in mouse Atg4b was previously shown to inhibit autophagy [28]. Using the GFP-Lc3 assay, we showed that expression of zebrafish Atg4bC74A significantly impairs GFP-Lc3 puncta formation (Supplementary Figure 2). We crossed *Tg(mitfa:atg4b^C74A^)* to *tp53^M214K/M214K^* homozygotes to generate *Tg(mitfa:atg4b^C74A^)*; *tp53^M214K/+^* heterozygotes and non-transgenic *tp53^M214K/+^* heterozygous siblings. 5/48 (10.4%) of *Tg(mitfa:atg4b^C74A^)*; *tp53^M214K/+^* heterozygotes developed tumors with a median tumor onset of 12.7 months. In contrast, none of the *tp53^M214K/+^* heterozygous siblings (*n* = 48) had developed tumors by 18 months of age at which point the experiment was stopped (not shown). Thus, these data confirm that inhibition of autophagy decreases the latency of tumor onset in *tp53^M214K/+^* heterozygotes.

### Tumor spectrum of zebrafish expressing Tg(*mitfa:atg5^K130R^*) in the *tp53* mutant background

**Figure 3:**
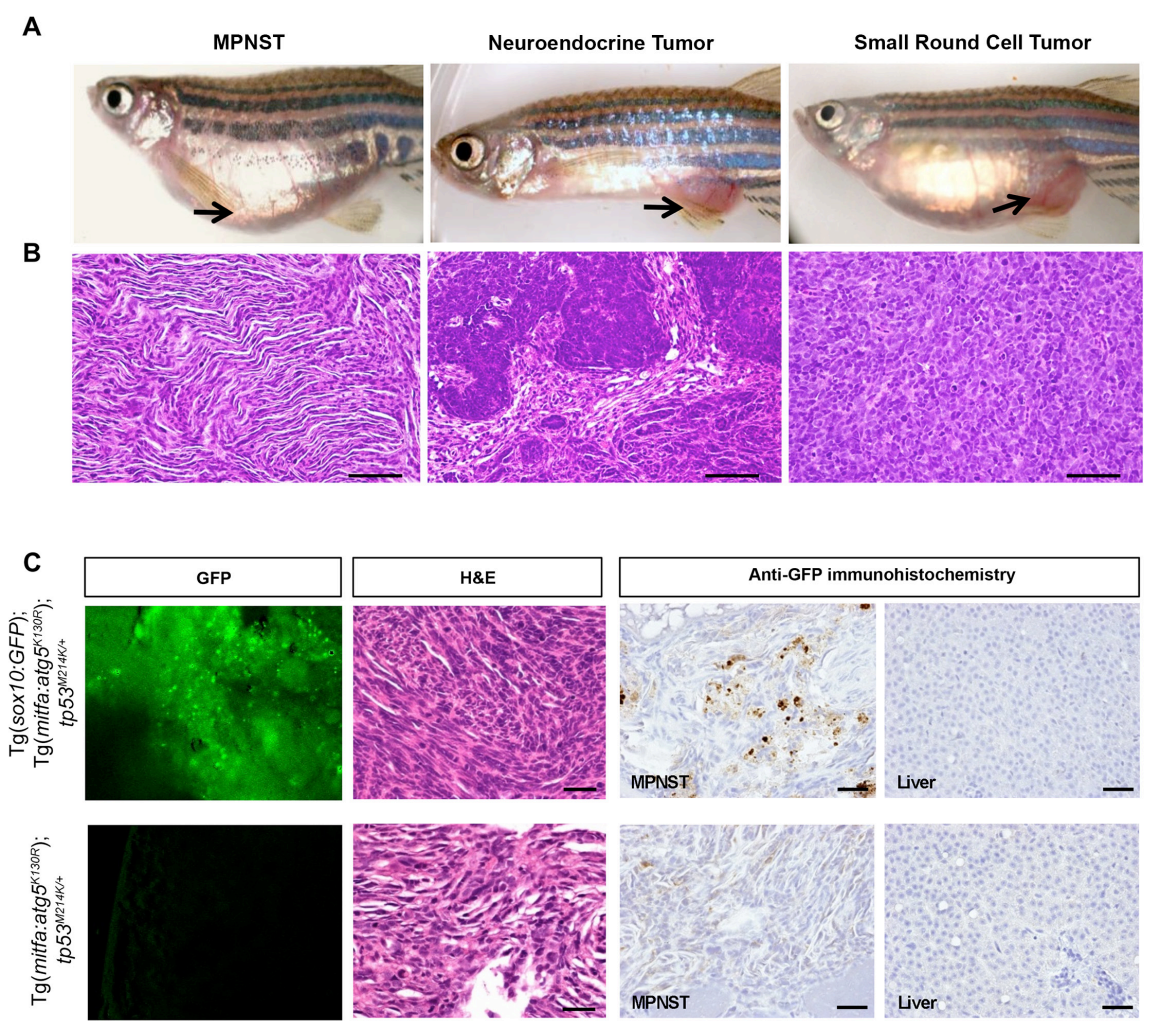
MPNSTs arising in Tg(*mitfa:atg5^K130R^*); *tp53^M214K/+^ fish* express sox10 **A.**-**B.** Representative photomicrographs of gross macroscopic appearance of tumors **A.** and hematoxylin and eosin (H&E) stained microscopic sections of corresponding tumors **B.** in Tg(*mitfa:atg5^K130R^*)*; tp53^M214K/+^*fish. MPNST, malignant peripheral nerve sheath tumor. Scale bars, 50 μm. See Table 1 for details of numbers of fish with each tumor type. **C.** Representative confocal images to detect GFP expression, H&E staining, and anti-GFP immunoperoxidase staining of an MPNST from a Tg(*mitfa:atg5^K130R^*)*;* Tg(*sox10:EGFP*)*; tp53^M214K/+^* fish (top) and an MPNST from a GFP-negative control Tg(*mitfa:atg5^K130R^*)*; tp53^M214K/+^* (bottom). Liver is shown for the anti-GFP immunoperoxidase staining as a representative non-tumor tissue (right panels). Scale bars, 100 μm.

Macroscopically, tumors predominantly occurred in the abdominal region (Figure 3A) and were characterized by the presence of prominent blood vessels on the ventral surface. A small number of tumors also arose behind the eyes and in the jaw and the gills (data not shown). The most common tumors were MPNSTs, characterized by wavy fascicles of spindle cells with elongate nuclei showing tapered ends. The next most common tumors were characterized by cords, nests, and gland-like structures composed of epithelioid cells with high nuclear to cytoplasmic ratio, hyperchromatic nuclei, and scant eosinophilic cytoplasm, morphologically resembling human poorly differentiated neuroendocrine carcinomas. Five of the Tg(*mitfa:atg5^K130R^*);*tp53^M214K/+^* fish and one Tg(*mitfa:atg5^K130R^*);*tp53^M214K/M214K^* fish developed malignant small cell tumors, which contained sheets of small round cells with finely dispersed chromatin, high nuclear-cytoplasmic ratio and scant eosinophilic cytoplasm. Small cell tumors were not found in non-transgenic *tp53^M214K/+^* or *tp53^M214K/M214K^* controls (Figure 3A, 3B, and Table 1).

**Table 1:** Tumor histology in different genetic backgrounds

*p53* Genotype	+/+		M214K/+		M214K/M214K	
*mitfa:atg5^K130R^* transgene	-	+	-	+	-	+
Total # per genotype	48	84	94	150	66	54
Total # with tumor (%)	0	0	14 (15%)	61 (40%)	39 (58%)	36 (67%)
MPNST	0	0	11	43	26	26
Neuroendocrine cell tumor	0	0	3	5	3	2
Small round cell tumor	0	0	0	5	0	1
Vascular tumor	0	0	0	0	2	0
Not determined	0	0	0	4	8	7

### Tg(*mitfa:atg5^K130R^*) MPNSTs express *atg5^K130R^*and *sox10*

Tg(*mitfa:atg5^K130R^*);*tp53^M214K/+^* fish showed a significantly higher tumor incidence than *tp53^M214K/+^* heterozygous mutant fish. However, both fish lines developed MPNSTs (Table 1), which was somewhat surprising for the Tg(*mitfa:atg5^K130R^*) fish, because expression of activated mutant *BRAF^V600E^* from the *mitfa* promoter was previously shown to cause melanomas in zebrafish [26]. To confirm that the *atg5^K130R^* transgene was expressed in tumors arising in Tg(*mitfa:atg5^K130R^*); *tp53^M214K/+^* fish, we isolated tumors, disaggregated the cells and monitored cell growth in primary cell cultures for up to 3 passages to obtain homogeneous cells. We isolated RNA from the cells and performed RT-PCR analysis to detect *atg5* mRNA. PCR products were confirmed by sequence analysis and showed the expression of *atg5^K130R^* RNA in tumor tissue (Supplementary Figure 2). We then sought further confirmation that these tumors indeed arose from a neural lineage. Specifically, we asked whether the tumors expressed markers of neural crest, which gives rise to Schwann cells [37-39], the cell of origin of MPNSTs [40, 41]. Expression of *mitfa* is regulated by sox10, a transcription factor expressed in neural crest cells [42] and re-expressed during melanoma initiation [43]. We crossed Tg(*mitfa:atg5^K130R^*);*tp53^M214K/+^* fish to the neural crest cell reporter fish line, Tg(−4.9*sox10:GFP*) [44] and monitored the fish for tumor development. A representative tumor arising in this line is shown in Figure 3C. The tumor expressed GFP as detected by confocal microscopy (Figure 3C, first column). Histologically, the tumor was identified as MPNST (Figure 3C, second column) and GFP expression in tumor tissue was confirmed by immunostaining (Figure 3C, third column). GFP expression was variable; some areas of the tumor showed strong expression of GFP, but spindle-like tumor cells showed very weak or almost negative expression of GFP, likely caused by loss of *sox10* promoter activity in fully differentiated cells [45]. GFP expression was not detected in non-tumorigenic liver (Figure 3C, fourth column). These results confirm that MPNSTs in Tg(*mitfa:atg5^K130R^*);*tp53^M214K/+^* fish arise from neural crest cells and suggest that impairment of autophagy in this lineage increases tumor susceptibility.

### Cell proliferation rates are equivalent in MPNSTs from *atg5^K130R^* transgenic and non-transgenic *tp53^M214K/+^* heterozygotes

**Figure 4:**
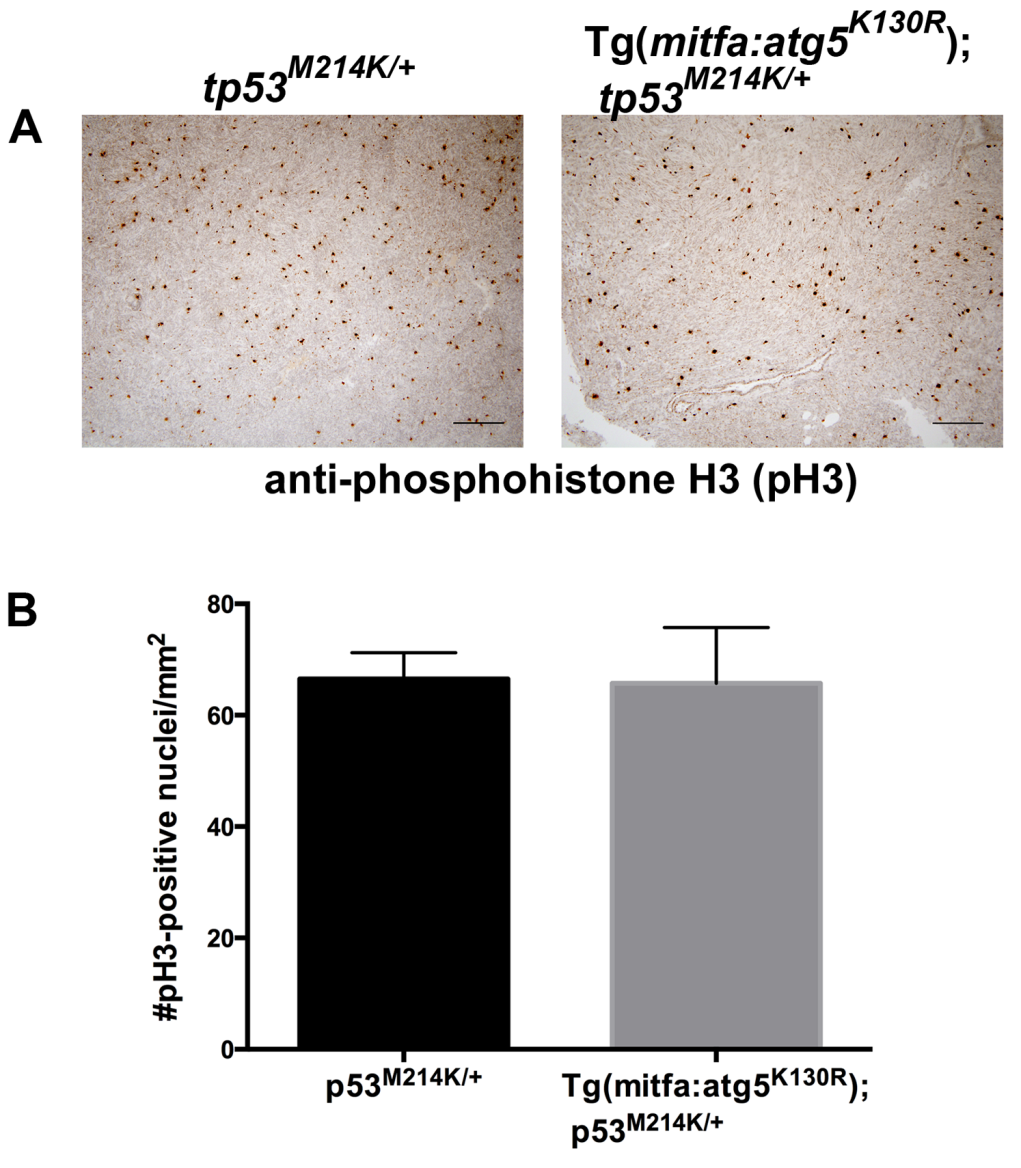
Cell proliferation rates are equivalent in MPNSTs from *atg5^K130R^* transgenic and non-transgenic p53-deficient fish **A.** Anti-phosphohistone H3 immunohistochemistry of *tp53^M214K/+^* and Tg(*mitfa:atg5^K130R^*)*; tp53^M214K/+^* fish. **B.** Quantification of staining. Mean +/− SEM of 5 tumors per genotype is shown.

MPNSTs arising in Tg(*mitfa:atg5^K130R^*);*tp53^M214K/+^* fish were grossly evident much earlier than similar tumors occurring in *tp53^M214K/+^* heterozygous mutant fish. This raised the possibility that tumors in *atg5^K130R^* transgenics might undergo more rapid cell proliferation than those in non-transgenic *tp53^M214K/+^* heterozygotes, making the tumors detectable earlier. To test this possibility, we prepared histologic sections of MPNSTs from *atg5^K130R^* transgenic and non-transgenic *tp53^M214K/+^* heterozygotes and performed immunohistochemistry for the Serine 10-phosphorylated form of Histone H3, a marker of mitotic cells [46]. MPNSTs from both *atg5^K130R^*-transgenic and non-transgenic animals exhibited similar levels of cell proliferation (Figure 4), suggesting that the early appearance of MPNSTs in *atg5^K130R^*-transgenic p53 heterozygotes is not due to intrinsically higher rates of cell proliferation in these tumors.

### MPSNTs arising in Tg(*mitfa:atg5^K130R^*) fish have increased double-stranded DNA breaks

**Figure 5:**
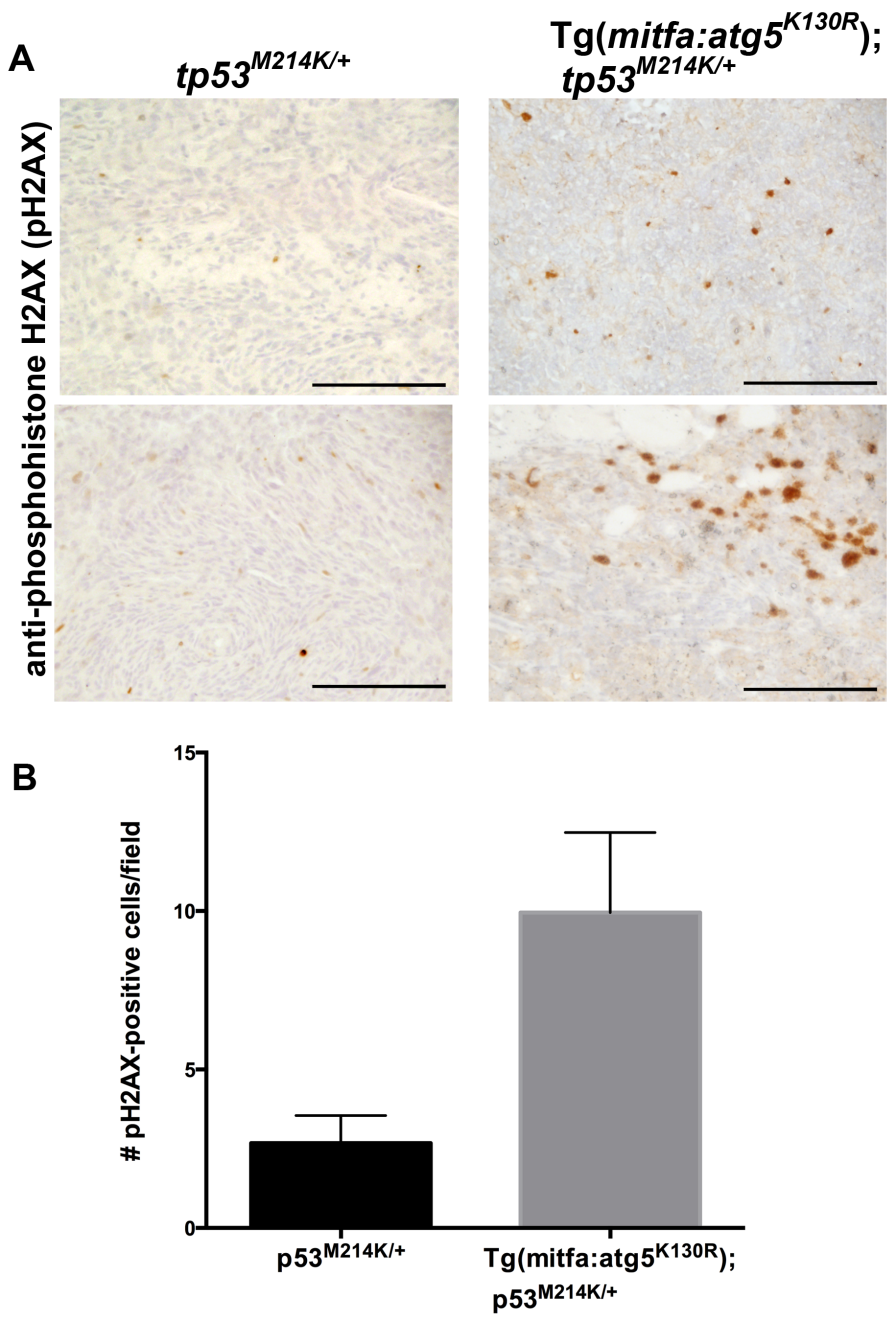
MPSNTs arising in *atg5^K130R^* transgenics have increased double-stranded DNA breaks **A.** Anti-phosphohistone H2AX immunohistochemistry of *tp53^M214K/+^* and Tg(*mitfa:atg5^K130R^*)*; tp53^M214K/+^* fish. Scale bar: 100 μm. **B.** Quantification of staining. Mean +/− SEM of 40 fields from each of 5 tumors per genotype is shown.

To further probe the mechanism of increased tumor incidence in Tg(*mitfa:atg5^K130R^*);*tp53^M214K/+^* fish, we considered whether inhibition of autophagy in neural crest lineage cells caused genome instability. Previous studies have shown that cells deficient in autophagy exhibit chromosome instability manifested by increased DNA damage, gene amplification, and aneuploidy [47, 48]. Irradiated glioblastoma cells in which autophagy is inhibited displayed more pronounced and prolonged foci of phosphohistone H2AX, a marker of DNA double-strand breaks [49]. To test whether inhibition of autophagy caused elevated levels of DNA damage in the transgenic fish, we stained MPNSTs from Tg(*mitfa:atg5^K130R^*);*tp53^M214K/+^* and *tp53^M214K/+^* heterozygous mutants with a validated antibody specific for the phosphorylated form of Histone H2AX (pH2AX) [50]. Tumors arising in fish with the *mitfa:atg5^K130R^* transgene exhibited significantly higher numbers of pH2AX foci (Figure 5). Thus, inhibition of autophagy was associated with increased generation and/or persistence of double-strand DNA breaks in Tg(*mitfa:atg5^K130R^*);*tp53^M214K/+^* tumors.

### Inhibition of autophagy promotes *tp53* loss-of-heterozygosity and tumorigenesis

**Figure 6:**
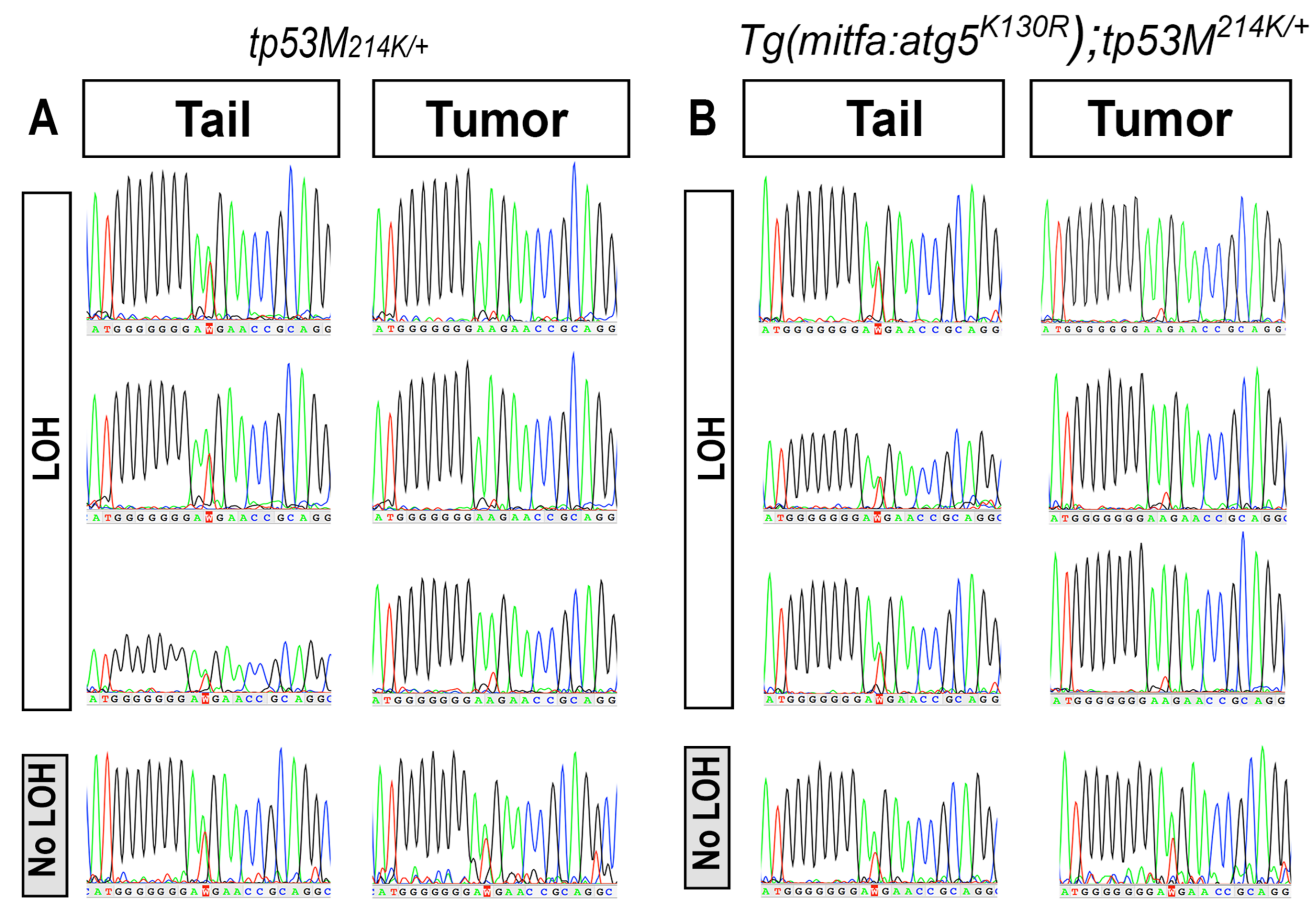
p53 loss of heterozygosity in MPNSTs arising in *atg5^K130R^* transgenic and non-transgenic *tp53^M214K/+^* heterozygotes.Genomic DNA was amplified from MPNST tumor and from non-tumor tail tissue from *tp53^M214K/+^* and Tg(*mitfa:atg5^K130R^*)*; tp53^M214K/+^* fish Representative sequence traces of a fragment of the *tp53* coding sequence harboring the M214K mutation are shown.

We did not observe tumors in fish that stably express Atg5^K130R^ in a *tp53* wild-type background. Since increased tumor incidence due to Atg5^K130R^ expression was only detected in a *tp53* heterozygous mutant background, we hypothesized that expression of Atg5^K130R^ might affect the function or expression of tp53. tp53 functions as a homotetramer in cells, and the defective apoptosis phenotype of heterozygous *tp53^M214K/+^* mutant fish was found to be intermediate compared to that of the homozygous *tp53^M214K/M214K^* mutant fish, implying that the total cellular level of p53 protein is functionally important [36]. We also noted that the tumor incidence in Tg(*mitfa:atg5^K130R^*);*tp53^M214K/+^* fish resembles that of p53 homozygous *tp53^M214K/M214K^* mutant (non-transgenic) fish, with a slightly later onset. *TP53* loss of heterozygosity (LOH) frequently occurs during the development of human cancers, including MPNSTs [51-55]. The elevated incidence of DNA damage in Tg(*mitfa:atg5^K130R^*);*tp53^M214K/+^* MPNSTs prompted us to test the incidence of *tp53* LOH in these tumors (Figure 6). Pair-matched tumor and normal tissue genomic DNA was prepared from the individual tumor-bearing fish, and a fragment of the *tp53* gene containing the M214K mutation was PCR-amplified and sequenced. LOH of *tp53* occurred in > 70% of tumors from both Tg(*mitfa:atg5^K130R^*);*tp53^M214K/+^* and non-transgenic *tp53^M214K/+^* fish [22/30 (73.3%) of tumors from Tg(*mitfa:atg5^K130R^*);*tp53^M214K/+^* and 7/10 (70%) of tumors in heterozygous tp*53^M214K/+^* fish (70%)] (representative sequences are shown in Figure 6). Given the significantly shortened latency of tumorigenesis in the Tg(*mitfa:atg5^K130R^*); *tp53^M214K^* fish, these data suggest that inhibition of autophagy by the expression of Atg5^K130R^ may accelerate LOH of p53. These results further suggest a possible role for autophagy in tumor suppression by regulating LOH of tumor suppressor genes.

## DISCUSSION

There is ongoing controversy regarding the role of autophagy in tumorigenesis (reviewed in [4, 13]). Autophagy may play a role in tumor suppression, as suggested by studies involving genetic deletion of autophagy proteins in mouse models. However, autophagy is also known to play a critical role in the survival of cells under stress, and therefore autophagy may promote the survival and growth of cancer cells. The data we present here suggest that inhibition of autophagy in a specific developmental lineage increases the incidence of cancer in that lineage, not through a direct oncogenic mechanism but rather by modulating a pre-existing cancer susceptibility, in this case due to tp53 deficiency.

Expression of dominant-negative proteins as a strategy to inhibit autophagy has not previously been reported in zebrafish; therefore, we took several steps to validate this approach. We showed by immunoblot analysis and using a live autophagy reporter line that expression of *atg5^K130R^* inhibits autophagy *in vivo*. We further showed that the transgene is expressed in tumors arising in transgenic fish.

Previous studies employing the zebrafish *mitfa* promoter to drive mutated human *BRAF^V600E^* or *NRAS^Q61K^* have documented a high incidence of melanomas in the transgenic fish [26, 56]. Notably, in both these cases, melanomas only arose in the context of *tp53* deficiency. One study reported that *ATG5* is often downregulated in human melanomas compared to preneoplastic nevi, and that low *ATG5* expression is an adverse prognostic indicator in melanoma [57]. The same study demonstrated that the autophagy pathway appears to serve as a barrier to oncogene-induced senescence in melanocytes expressing mutant *BRAF*. In our study, however, Tg(*mitfa:atg5^K130R^*) zebrafish displayed normal pigmentation and failed to develop nevi or melanomas, in either wildtype and p53-deficient backgrounds. Taken together with our data, these results suggest that the tumor-promoting effects of deficient autophagy depend on specific cellular contexts. In melanocytes, deficient autophagy may contribute to the development or progression of melanoma, but is likely not sufficient to cause melanoma in the absence of a *RAS* or *BRAF* activating mutation.

While we did not detect melanomas, Tg(*mitfa:atg5^K130R^*);*tp53^M214K/+^* and Tg(*mitfa:atg5^K130R^*); *tp53^M214K/M214K^* lines developed several different types of tumors, most commonly MPNSTs and neuroendocrine tumors. This tumor spectrum is consistent with the developmental origins of *mitfa*-expressing cells. In zebrafish, as in other vertebrates, neural crest cells give rise to peripheral neurons, glia, Schwann cells and melanocytes during migration from the dorsal neural tube into target tissues [58, 59]. More recently, a second possible cell of origin for melanocytes has been identified, namely Schwann Cell Precursors (SCPs) in peripheral nerves [60]. While we cannot rule out the possibility that dominant-negative autophagy protein expression driven by the *mitfa* promoter alters cell fate determination of cranial neural crest cells, resulting in aberrant Schwann cell development and MPNSTs, it is more likely that inhibition of autophagy affects the development of SCPs and promotes tumorigenesis. In this setting, expression of *atg5^K130R^* and inhibition of autophagy may prevent normal development of melanocytes from migrating SCPs. Indeed, Adameyko *et al*. showed that Sox10+/Krox20- SCPs acquire Mitf expression as cells migrate away from peripheral nerves [60]. A recent study from Kaufman and Zon and colleagues showed that expression of the embryonic marker *crestin* in neural crest progenitors is driven by Sox10 together with the Mitf and Tfap2 transcription factors. In a model expressing activated mutant BRAF^V600E^, re-expression of *crestin* marked the earliest events in melanoma formation, and overexpression of Sox10 accelerated the formation of melanomas [43]. Interestingly, 49% of human MPNSTs express SOX10 [61]. However, we note that MPNSTs also arise in *tp53*-deficient animals in the absence of the atg5K^130R^ transgene, and thus these results do not preclude haploinsufficiency of *tp53* itself, as opposed to a direct effect of impaired autophagy in Schwann cell precursors, may be the main driving force in the development of MPNSTs.

Another possibility is that the *mitfa* promoter fragment used in these studies (∼5 kb of genomic DNA) does not perfectly recapitulate the activity of the endogenous *mitfa* locus, leading to ectopic transgene expression in peripheral nerve tissues. However, the development of non-MPNST neural tumors such as neuroendocrine and small-cell tumors in the transgenic line suggests that autophagy inhibition acts directly on a neural precursor cell and mediates or facilitates tumorigenesis. In this regard, it is interesting to note that the PI3-Kinase mTOR pathway, which acts to inhibit the autophagy pathway [62, 63], has very recently been shown to contribute to the pathogenesis of both MPNSTs and neuroendocrine tumors, the two most common tumor types manifested by Tg(*mitfa:atg5^K130R^*);*tp53^M214K/+^* fish [64, 65]. Our studies, however, do not directly test the possible role of the PI-3 kinase pathway in zebrafish MPNSTs, and further experiments will be required to distinguish these possibilities.

Several zebrafish cancer models demonstrate a propensity to develops MPNSTs, including those harboring mutations in tp53, ribosomal proteins or mismatch repair proteins, or mutations causing genomic instability [36, 66-69]. These mutant lines share the common characteristic of genomic instability. In this regard, it is particularly interesting that inhibition of autophagy has also been reported to cause genomic instability [47]. We demonstrated increased levels of DNA double-strand breaks in MPNSTs in the Tg(*mitfa:atg5^K130R^*);*tp53^M214K/+^* background. Thus the mechanism of accelerated MPNST development in fish with impaired autophagy may be increased genomic instability. However, our experiments do not definitively establish that impaired autophagy, and not another cause, is responsible for the excess DNA damage in the MPNSTs arising in Tg(*mitfa:atg5^K130R^*);*tp53^M214K/+^* fish.

Consistent with this hypothesis, expression of the *atg5^K130R^* transgene greatly accelerated the development of tumors in *tp53^M214K+^* heterozygous mutants. Berghmans *et al.* reported tumor incidence only in homozygous mutant *tp53^M214K^* fish [36], whereas in our studies, *p53* heterozygous mutant fish also developed MPNSTs after 16 months of age. The inconsistency in tumor phenotypes may due to the late onset of tumors in the heterozygous *tp53* mutant fish line, arising after 16 months of age, beyond the age range reported by Berghmans *et al.* The relatively long latency of tumor development in *tp53^M214K/+^* heterozygotes suggested that biallelic mutation of p53 is necessary for tumor development. To address this hypothesis, each tumor bearing-fish was analyzed for its p53 status. Strikingly, more than 70% of tumors showed LOH of *tp53*, both in the Tg(*mitfa:atg5^K130R^*) heterozygous *tp53 ^M214K^* mutant background or the non-transgenic heterozygous *tp53 ^M214K/+^* mutant line, suggesting LOH may be the central route whereby tumors arise in a *tp53^M214K/+^* background. In zebrafish cancer models of *tp53* deficiency, LOH of *tp53* has not been reported except in the *tp53^I166T^* mutant fish line [70]. Here, we showed that expression of Atg5^K130R^ did not increase the frequency of LOH but clearly accelerated LOH of p53 in a *tp53^M214K/+^* background and led to enhanced tumor development. The precise mechanisms of increased tumor susceptibility are not yet clear, but may relate to genomic instability caused by impaired autophagy. Of relevance, a recent study demonstrated that the contribution of autophagy to tumor susceptibility in a Kras mouse model of pancreatic cancer depends on the p53 status [22]. In the presence of wildtype p53 levels, loss of autophagy impaired tumor progression. In contrast, in the absence of p53 function, loss of autophagy greatly accelerated the onset of pancreatic carcinoma. This raises the possibility that impaired autophagy may contribute to tumorigenesis in at least two ways: first by promoting genomic instability and LOH of *tp53*, and then by providing a selective advantage to the growth of tp53-deficient cells.

## MATERIALS AND METHODS

### Zebrafish strains and maintenance

Fish were raised and maintained under standard conditions [71]. Embryos from the wild type AB strain were used for injection to generate the Tg(*mitfa:atg5^K130R^*) line. All animals crossed with wild type AB, the autophagy reporter line Tg(*cmv:GFP-lc3*) [31]*,* or the zebrafish *p53* mutant line *tp53^M214K^* [36] and maintained. Autophagy reporter fish Tg(*cmv:GFP-lc3*) was were obtained from Dr. Daniel Klionsky (University of Michigan). Tg (*-4.9sox10:eGFP*) zebrafish were kindly provided by Dr. Tom Schilling (UC-Irvine). *Tp53^M214K^* mutant zebrafish were a gift of Dr. A. Thomas Look (Dana-Farber Cancer Institute). All animal protocols were approved by the UT Southwestern Medical Center Institutional Animal Care and Use Committee.

### *In vitro* transcription and RNA injection

To construct a dominant-negative version of zebrafish *atg5,* zebrafish cDNA was prepared from 24 hpf embryos. Amplified *atg5* cDNA was cloned into the pGEM-T Easy vector (Promega) and mutated using the Quick Change XL Site-Directed mutagenesis kit (Stratagene) to generate the K130R mutation. mCherry cDNA was amplified using pME-mCherry (kindly provided by Dr. Nathan Lawson, University of Massachusetts Medical Center) and cloned into pGEM-T Easy vector. Constructs were verified for their sequences before *in vitro* transcription. Capped mRNAs used for injection were prepared by *in vitro* transcription using mMessage mMachine kit (Ambion, AM1340). *In vitro* transcribed RNAs were injected into one-cell stage embryos.

### Zebrafish embryonic cell culture

Approximately 50 embryos injected with *atg5*, *atg5^K130R^* or mCherry mRNA or with *atg5* morpholino were used for primary cell culture. Injected embryos at the shield stage were sterilized with 70% ethanol, and dissociated in trypsin/EDTA solution. Primary cells were plated on a multi-chamber slide (Nunc) in LDF (Leibovitz 50%, DMEM 35%, F-12 15%) medium containing antibiotics and incubated at 25°C for 18 hours. Cells were imaged with epifluorescence microscopy and the number of GFP-LC3 dots/cell was quantified.

### Western blot analysis

Single cell-stage embryos were injected with 200 pg mRNA encoding *mCherry* (control), zebrafish *atg5*, or dominant-negative *atg5^K130R^* and primary cell cultures were prepared and used for immunoblotting. Cells were harvested and incubated for 30 min on ice in lysis buffer containing 150mM NaCl, 10mM Tris pH7.4, 0.2% Triton X-100, 0.3% NP-40, 0.2mM Na_3_VO_4_ and protease inhibitors (Roche). After centrifugation at 13,000 x *g*, supernatants were used for imunoblotting. Atg5 antibody (Novus, NB110-53818), LC3 antibody (Novus, NB100-2220) and β-actin antibody (Millipore, MAB1501R) were used for immunoblotting.

### Microscopic imaging

Images of GFP-Lc3 in primary cells were acquired on a Zeiss LSM 510 META confocal microscope. Primary cells were fixed in 4% PFA for 30 min and mounted using ProLong Gold antifade reagent (Invitrogen, P36935). Images of whole-mount zebrafish were taken with a Nikon Coolpix 4500 camera mounted on a Leica MZ125 stereo dissecting microscope.

### Cloning of expression constructs for transgenic line generation

Transgenesis constructs were assembled using Gateway recombinational cloning (Invitrogen) as previously described [72]. Transgenic zebrafish lines were generated using the *Tol2*-system as described [34]. Supplementary Table 1 lists the primers used. All expression constructs were verified for their sequences before use.

### Transgenic fish generation, screening and genotyping

Expression constructs (50 ng/μL) with transposase mRNA (100 ng/μL) were injected into one cell stage-embryos. Injected embryos were raised to adulthood and in-crossed for transgenic screening. Pools of embryos were screened for transgene incorporation by PCR reactions. Putative founders were out-crossed for further screening and transgenic F1 adults were identified by tail clipping and genomic PCR reactions.

### Zebrafish RNA preparation and RT-PCR

To confirm the expression of transgenes in transgenic fish lines, RNA was extracted from Tg(*mitfa:atg5^K130R^*) tumor tissue or cultured primary tumor cells using Trizol reagent (Invitrogen) according to the manufacturer's instructions. cDNAs were synthesized using 5 μg of purified RNAs by RT^2^ HT first-strand kit (Qiagen). PCR reactions and sequencing analysis were performed to detect endogenous or transgenic *atg5* transcripts.

### Histological analysis

Zebrafish were euthanized with 50% Tricaine solution, fixed in 4% PFA for 48 h and decalcified for 5 days in 0.5M EDTA. Tumor sections were stained with H&E for tissue pathological diagnosis.

### Primary cell culture of zebrafish MPNSTs

Tumors were dissected from euthanized zebrafish. Tissues were washed twice in PBS containing antibiotics (Gibco), and digested for 30 min at 37°C using dispase (Becton Dickinson). Digested tissues were washed twice in PBS, plated on a multi-chamber slide (Nunc) in LDF (Leibovitz 50%, DMEM 35%, F-12 15%) medium containing antibiotics, and incubated at 25°C. After 3 passages, cells were harvested and used for RT-PCR.

### Assessment of tumor incidence

To generate cohorts of animals for the tumor study, *Tg(mitfa:atg5^K130R^)* heterozygotes (all derived from the same founder) were crossed to *tp53^M214K/+^* heterozygotes to generate *Tg(mitfa:atg5^K130R^)*; *tp53^M214K/+^* animals. These were backcrossed to *tp53^M214K/+^* heterozygotes to generate the following genotypes: 1) non-transgenic, *tp53^+/+^*; 2) non-transgenic, *tp53^M214K/+^*; 3) non-transgenic, *tp53^M214K/M214K^*; 4) *Tg(mitfa:atg5^K130R^), tp53^+/+^*; 5) *Tg(mitfa:atg5^K130R^)*, *tp53^M214K/+^*; 6) *Tg(mitfa:atg5^K130R^)*, *tp53^M214K/M214K^*. Animals were raised in groups of 40 fish per 9L tank on a single aquarium system and monitored daily. The end-point for all animals was the first appearance of tumor. Once a tumor was detected, animals were sacrificed. Tail tissue was taken for genotyping for the atg5K130R transgene and the p53 M214K mutation. Tumor tissue was divided; part of the tumor was fixed and submitted for histologic analysis and the remainder was flash-frozen and preserved for molecular analysis. At 24 months of age all remaining tumor-free animals were sacrificed and genotyped. Tumor incidence was analyzed statistically using the log-rank test.

### Immunohistochemistry

For immunostaining, slides were deparaffinized and antigen retrieval was performed for 15 min in Trilogy reagent (Cell Marque). After quenching peroxidase activity and blocking nonspecific binding, slides were incubated for 1 h with anti-GFP antibody (MBL), anti-phosphohistoneH3 Serine 10 antibody (Santa Cruz Biotechnology) or anti-phosphohistone H2AX [50] followed by incubation with HRP-conjugated anti-rabbit antibody (Immpress kit, Vector Labs) for 30 min in a wet chamber. Slides were developed with DAB solution, counterstained with hematoxylin (Invitrogen), dehydrated, and mounted with Permount mounting media (Fisher).

## SUPPLEMENTARY MATERIALS FIGURES


