# Supplementary material for: Genetic inhibition of autophagy promotes p53 loss-of-heterozygosity and tumorigenesis
Source: Oncotarget. 2016 Sep 16;7(42):67919–33. doi: 10.18632/oncotarget.12084 (PMC5356529; doi:10.18632/oncotarget.12084)
Supplement: Supplementary file 1 [file oncotarget-07-67919-s001.pdf]

Genetic inhibition of autophagy promotes p53 loss-of-heterozygosity and tumorigenesis

Supplementary Material

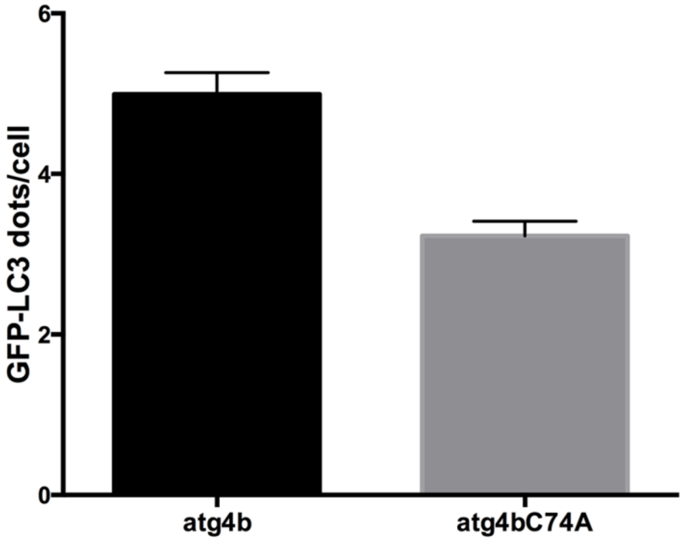

Supplementary Figure 1. Expression of dominant-negative atg4b impairs autophagy in zebrafish. Approximately 50 embryos injected with *atg4b*^{C74} or mCherry mRNA were used for primary cell culture. Primary cells were incubated for 18 hours. Cells were imaged with epifluorescence microscopy and the number of GFP-Lc3 dots/cell was quantified. Bars show mean +/- SEM for n=100 cells/condition.

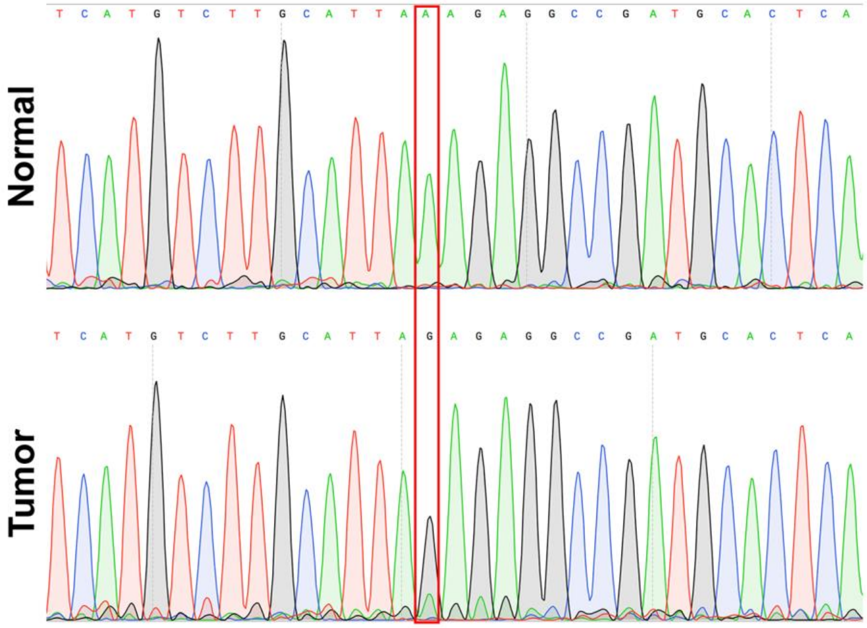

Supplementary Figure 2. Representative sequence analysis of cDNA derived from an MPNST tumor in a Tg(*mitfa:atg5*^{K130R})*; tp53*$^{M214K/+}$ fish showing the A>G mutation in dominant negative *atg5* at nucleotide 389 (red box). Wildtype *atg5* sequence is shown for comparison.
